# GENDER DIFFERENCES IN SKELETAL MUSCLE ENERGETICS EXPLAIN THE MOBILITY IMPAIRMENT DISPARITY IN SOMMA

**DOI:** 10.1093/geroni/igad104.2082

**Published:** 2023-12-21

**Authors:** Philip Kramer, Giovanna Distefano, Sofhia Ramos, Bret Goodpaster, Anne Newman, David Marcinek, Peggy Cawthon, Anthony Molina

**Affiliations:** Wake Forest University School of Medicine, Winston-Salem, North Carolina, United States; AdventHealth, Orlando, Florida, United States; AdventHealth, Orlando, Florida, United States; AdventHealth, Orlando, Florida, United States; University, Pittburgh, Pennsylvania, United States; University of Washington, Seattle, Washington, United States; California Pacific Medical Center, Research Institute, San Francisco, California, United States; University of California, San Diego, San Diego, California, United States

## Abstract

An age-related decline in muscle mitochondrial energetics is known to contribute to the loss of muscle function in older adults. Women experience a higher prevalence of mobility impairment compared to men, but it is unknown whether gender-specific differences in muscle energetics underlie this disparity. In the Study of Muscle, Mobility and Aging (SOMMA), muscle energetics were characterized using in vivo 31-Phosphorus Magnetic Resonance Spectroscopy and High-Resolution Respirometry of the vastus lateralis. A Short Physical Performance Battery score ≤ 8 was used to define lower-extremity mobility impairment. In this analysis of 773 participants age 70-94, women had greater odds (OR=1.83, p=0.03) of mobility impairment compared to men, which was largely explained by significantly lower muscle energetics (Maximal OXPHOS in Women= 55.06 +/- 15.95; Men= 65.80 +/- 19.74; p<0.0001) using mediation modeling (adjusted OR=1.25, p=0.45). The mobility impairment disparity could be further explained by BMI, race, physical activity, and number of comorbidities (adjusted OR=0.98, p=0.96). However, none of these covariates, including age, fully explained gender differences in muscle mitochondrial energetics (e.g. Adjusted Maximal OXPHOS in Women= 56.5, 95%CI=54.8, 58.1; Men= 64.4, 95%CI=62.5, 66.2; p<0.0001). Notably, high BMI and low gait speed were associated with lower muscle energetics, though BMI slightly more so in women (ATPmax BMI/gender interaction, p= 0.02) than men, and age was only significantly negatively associated with muscle energetics in men (e.g. Maximal ETS capacity age/gender interaction, p=0.04). In conclusion, women had lower muscle mitochondrial energetics compared to men, which largely explained their greater odds of lower-extremity mobility impairment.

